# The L-shaped relationship between composite dietary antioxidant index and sarcopenic obesity in elderly adults: a cross-sectional study

**DOI:** 10.3389/fnut.2024.1428856

**Published:** 2024-09-02

**Authors:** He Wu, Xiyi Chen, Zhengqing Shi, Jieyu Liu, Ziqi Meng, Chenguo Zheng, Chongjun Zhou

**Affiliations:** ^1^Department of Emergency, The Second Affiliated Hospital and Yuying Children’s Hospital of Wenzhou Medical University, Wenzhou, China; ^2^Department of Cardiovascular and Thoracic Surgery, The Second Affiliated Hospital and Yuying Children's Hospital of Wenzhou Medical University, Wenzhou, China; ^3^Department of Anorectal Surgery, The Second Affiliated Hospital and Yuying Children's Hospital of Wenzhou Medical University, Wenzhou, China; ^4^Zhejiang Inheritance and Innovation Team of Traditional Chinese Medicine Devoting to the Diagnosis and Treatment of Anorectal Diseases, Wenzhou, China

**Keywords:** composite dietary antioxidant index, sarcopenic obesity, NHANES, diet, elderly

## Abstract

**Background:**

This study aimed to examine the associations of the Composite Dietary Antioxidant Index (CDAI) with sarcopenic obesity (SO) using the National Health and Nutrition Examination Survey (NHANES) database.

**Methods:**

Data were gathered from NHANES between 2001 and 2004. To examine the relationship between CDAI and the occurrence of SO, multiple logistic regression analyses were performed. Subgroup analyses were performed to demonstrate the stability of the results. Restricted cubic splines were utilized to examine the non-linear correlations.

**Results:**

A total of 2,333 elderly individuals were included in the study. In the multivariate logistic regression crude model, we revealed an odds ratio (OR) of 0.928 [95% confidence interval (CI), 0.891–0.965, *p* < 0.001] for the correlation between CDAI and SO. The ORs were 0.626 (95% CI, 0.463–0.842) and 0.487 (95% CI, 0.354–0.667) for CDAI tertiles 2 and 3, respectively (*p* for trend <0.001), after full adjustment. The subgroup analysis findings demonstrated a reliable and enduring connection between CDAI and SO across various subgroups. However, the strength of the correlation between CDAI and SO was significantly affected by diabetes (*p* for interaction = 0.027). Moreover, restricted cubic spline analysis revealed an L-shaped relationship.

**Conclusion:**

The present study identified an L-shaped correlation between CDAI and SO in elderly participants’ demographics. The implications of these findings were significant for future studies and the formulation of dietary guidelines.

## 1 Introduction

SO refers to a medical and functional state where obesity, marked by an abundance of fat tissue, coexists with sarcopenia (1). As individuals progress in age, they commonly encounter a decrease in both muscle mass and functionality, frequently accompanied by an augmentation in adipose tissue. This progression heightens the probability of developing and encountering the consequences of SO (2). From a medical perspective, SO has the potential to result in the combined risk associated with the two distinct phenotypes of body composition (3). The adverse clinical outcomes of SO are extremely significant. Up to this point, different definitions and diagnostic constructs have been used to identify SO. However, it has consistently been shown to be a powerful and separate risk factor for frailty, comorbidities, and mortality in numerous common disease conditions. Additionally, it is associated with higher mortality rates, particularly in the older population (4, 5).

The CDAI, as formulated by Wright et al. (6), serves as a comprehensive measure encompassing various dietary antioxidants, thereby reflecting an individual’s overall dietary antioxidant intake profile. The construction of CDAI was based on their combined anti-inflammatory effect (7). Furthermore, due to their crucial significance in the majority of global diets, there is a growing concern regarding the impact of total antioxidant capacity on health. Earlier research discovered a negative correlation between CDAI and conditions such as osteoporosis, depression, muscle strength, chronic kidney disease, heart failure, and cardiovascular mortality (8–13).

The efficacy of dietary antioxidants as interventions for mitigating adverse health effects, such as oxidative stress and chronic inflammation, has been established. However, the relationship between CDAI and SO remains uncertain. This study aims to examine the independent and combined associations of CDAI with SO using the NHANES database.

## 2 Materials and methods

### 2.1 Study population

**Figure 1:**
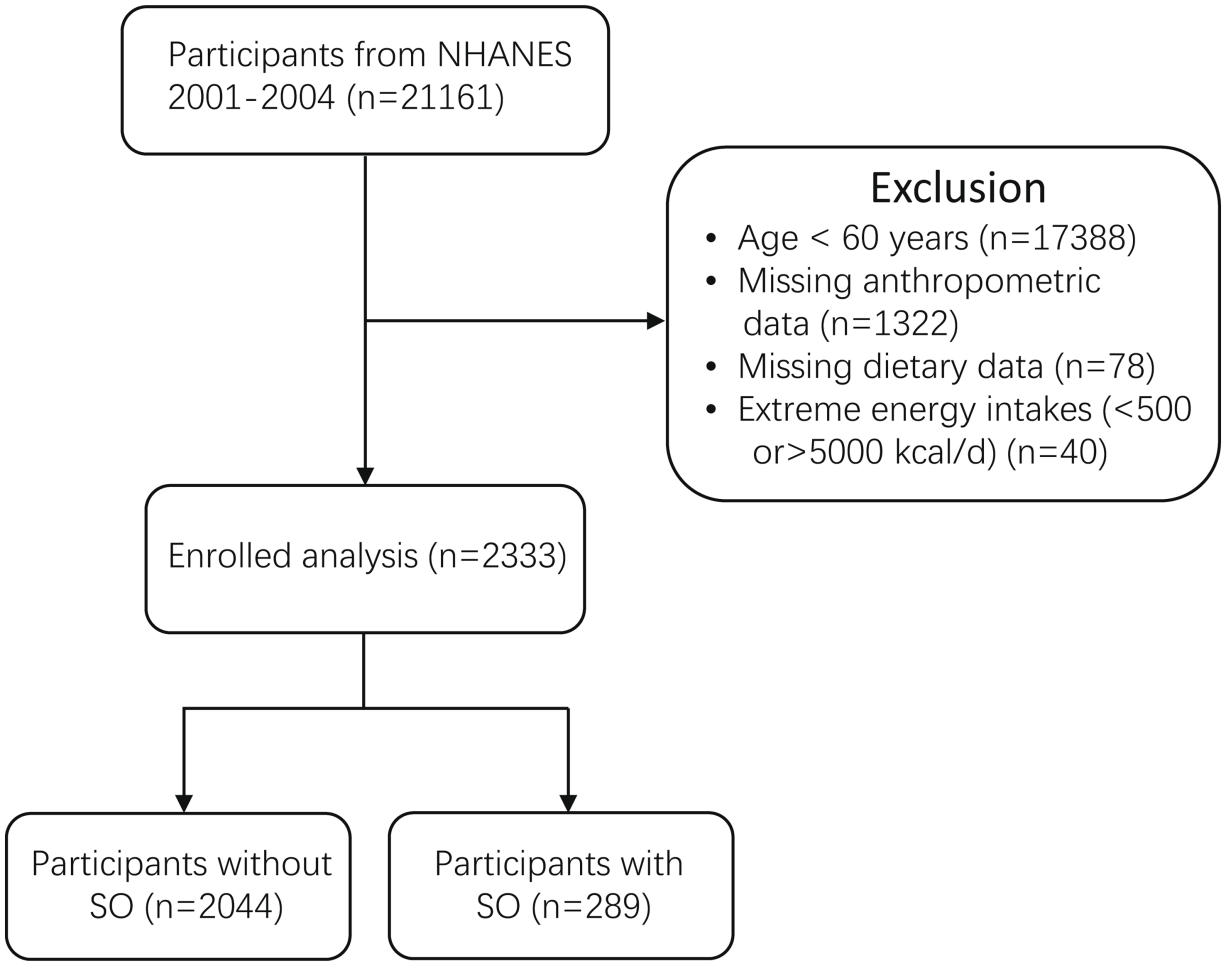
Flow chart of study participants.

The NHANES database, conducted by the Center for Disease Control and Prevention (CDC), was a nationally conducted cross-sectional study that sought to assess the health and nutritional status of non-institutionalized residents in the United States. Access to the survey data was available to researchers through online means. The National Center for Health Statistics (NCHS) ethical review board approved the study protocols. Eligible researchers were permitted to utilize the database without the need for an application. Confidentiality measures have been implemented to ensure that all patient information remains anonymous. Additionally, participants had provided informed consent by signing permission forms. After strict inclusion and exclusion criteria, we included two cycles with 2,333 elderly participants (Figure 1).

### 2.2 Assessment of CDAI

Dietary intake information was obtained using the initial 24-h dietary recall method. To evaluate combined exposure from antioxidant consumption in the diet, we used a revised edition of the CDAI formulated by Maugeri et al. (14). It is the sum of dietary intakes of six antioxidants (vitamin A, vitamin C, vitamin E, zinc, selenium, and carotenoids) calculated by subtracting the mean and dividing the result by the standard deviation (SD). The formula is provided below:


$$\overset{n=6}{\underset{i=1}{\sum }}\frac{\mathrm{Individual}\;\mathrm{Intake}-\mathrm{Mean}}{SD}$$

### 2.3 Definition of SO

Body composition was assessed using dual-energy x-ray absorptiometry (DXA) for its efficiency, simplicity, and minimal radiation exposure. DXA scans of the entire body were conducted using a Hologic QDR-4500A fan-beam densitometer at the NHANES mobile examination center (MEC). Certified radiology technologists performed the DXA examinations, ensuring a high standard of quality control was upheld during data collection and scan analysis, which included a strict phantom scanning regimen.

The total muscle mass of all upper and lower extremity limbs was defined as appendicular lean mass (ALM). Appendicular lean mass index (ALMI) was calculated by dividing ALM by the height squared. Sarcopenia was characterized by the widely referenced Baumgartner definition (ALMI <7.26 kg/m^2^ for men and ALMI <5.45 kg/m^2^ for women) (15). Obesity was defined as total body fat of ≥40% for women or ≥ 30% for men (16). SO was defined as the presence of both sarcopenia and obesity.

### 2.4 Covariates

The potential confounders considered in the present study encompassed age, race (Non-Hispanic White, Non-Hispanic Black, Mexican-American, and others), marital status (married, never married, others), education (high school or above or below), body mass index (BMI), diabetes, hypertension, smoking status, and alcohol drinking status. Diabetes was operationally defined as any participant who self-reported having diabetes, having a fasting plasma glucose level of 126 mg/dL or higher, or a glycated hemoglobin level of 6.5% or higher. Hypertension was operationally defined as individuals taking antihypertensive agents, having a systolic blood pressure of 140 mmHg or higher, or a diastolic blood pressure of 90 mmHg or higher.

### 2.5 Statistical analysis

Demographic features were presented as the average (SD) or median (IQR) for continuous variables and the proportion (%) for categorical variables. Categorical variables were analyzed using the chi-square test, while continuous variables were analyzed using the Student’s t-test or Mann–Whitney test. The relationship between CDAI and SO was analyzed using the logistic regression model. Model 1 was basic, while model 2 incorporated additional variables including age, sex, race, marital status, and education. Model 3 incorporated additional variables such as diabetes, hypertension, and BMI, building upon the foundation of model 2. Subgroup analyses were conducted while accounting for confounding variables. Restricted cubic splines were used to investigate the non-linear correlations. Statistical significance was determined by a two-sided *p*-value of less than 0.05, and the analyses were conducted using the R Studio version (4.3.1).

## 3 Results

The flow chart for selection is shown in Figure 1. Table 1 shows the characteristics stratified by SO status. A total of 2,333 participants were included in this study, and 289 of them had SO. The average age of all participants was 70 years, and the majority of them were non-Hispanic (60.14%). The range of CDAI scores varied from −6.165 (pro-inflammatory) to +31.579 (anti-inflammatory). Additionally, participants with SO had a higher mean CDAI score compared to those without SO (0.11 vs. –0.75, *p* < 0.001). Participants with SO were more prone to being older and having a lower BMI.

**Table 1:** Baseline characteristics of participants.

Variables	Total (*n* = 2,333)	Non-SO (*n* = 2,044)	SO (*n* = 289)	*P*
Age, years, median (IQR)	70.00 (65.00, 77.00)	70.00 (64.00, 77.00)	75.00 (68.00, 81.00)	< 0.001^*^
Sex				0.366
Male	1,165 (49.94)	1,013 (49.56)	152 (52.60)	
Female	1,168 (50.06)	1,031 (50.44)	137 (47.40)	
Race				< 0.001^*^
Non-Hispanic White	1,403 (60.14)	1,204 (58.90)	199 (68.86)	
Non-Hispanic Black	341 (14.62)	335 (16.39)	6 (2.08)	
Mexican-American	460 (19.72)	393 (19.23)	67 (23.18)	
Others	129 (5.53)	112 (5.48)	17 (5.88)	
Marital status				0.891
Married	1,411 (60.48)	1,239 (60.62)	172 (59.52)	
Never married	72 (3.09)	62 (3.03)	10 (3.46)	
Others	850 (36.43)	743 (36.35)	107 (37.02)	
Education level				0.281
Below high school	880 (37.72)	780 (38.16)	100 (34.60)	
High school	549 (23.53)	471 (23.04)	78 (26.99)	
Above high school	904 (38.75)	793 (38.80)	111 (38.41)	
Smoking status				0.229
Never	1,062 (45.52)	933 (45.65)	129 (44.64)	
Former	974 (41.75)	843 (41.24)	131 (45.33)	
Current	23 (7.3)	6 (16.2)	17 (6.2)	
Drinking status				0.122
Never	463 (19.85)	396 (19.37)	67 (23.18)	
Former	687 (29.45)	596 (29.16)	91 (31.49)	
Current	1,183 (50.71)	1,052 (51.47)	131 (45.33)	
Hypertension				0.941
No	702 (30.09)	614 (30.04)	88 (30.45)	
Yes	1,631 (69.91)	1,430 (69.96)	201 (69.55)	
Diabetes				0.350
No	1769 (75.83)	1,543 (75.49)	226 (78.20)	
Yes	564 (24.17)	501 (24.51)	63 (21.80)	
BMI, kg/m^2^, mean (SD)	27.82 (5.00)	28.18 (5.18)	25.27 (2.11)	< 0.001^*^
BMI				
< 25 kg/m^2^	691 (29.66)	552 (27.03)	139 (48.26)	< 0.001^*^
25–30 kg/m^2^	982 (42.15)	837 (40.99)	145 (50.35)	
≥ 30 kg/m^2^	657 (28.20)	653 (31.98)	4 (1.39)	
ASMI, kg/m^2^, mean (SD)	7.11 (1.35)	7.27 (1.32)	5.91 (0.90)	< 0.001^*^
Total body fat, %, mean (SD)	35.97 (7.91)	35.58 (8.10)	38.72 (5.77)	< 0.001^*^
CDAI, mean (SD)	0.00 (3.72)	0.11 (3.73)	–0.75 (3.57)	< 0.001^*^
CDAI				< 0.001^*^
Tertile 1	778 (33.35)	650 (31.80)	128 (44.29)	
Tertile 2	778 (33.35)	691 (33.81)	87 (30.10)	
Tertile 3	777 (33.30)	703 (34.39)	74 (25.61)	

Values are shown as numbers (%) unless otherwise indicated. ^*^Statistically significant (*p* < 0.05). SD, Standard deviation; IQR, interquartile range; SO, sarcopenic obesity; BMI, body mass index; ASMI, Appendicular Skeletal Muscle Index, CDAI, Composite Dietary Antioxidant Index, CDAI Tertile rang: Tertile 1: −6.165, −1.914; Tertile 2: −1.913, 0.643; Tertile 3: 0.644, 31.579.

Table 2 shows the logistic regression analysis of the association between CDAI and SO. The relationship between CDAI and SO remained consistent across various models. In model 1, the OR of CDAI on SO was 0.928 [95% CI, 0.891–0.965]. Participants in the uppermost CDAI tertiles compared to those in the lowest CDAI tertiles had an increased susceptibility to SO [OR: 0.535 (95% CI, 0.392–0.723)]. In model 3, after adjusting for age, sex, race, marital status, education, smoking status, drinking status, hypertension, diabetes, and BMI, the OR was 0.921 (95% CI, 0.883–0.956). The ORs were 0.626 (95% CI, 0.463–0.842) and 0.487 (95% CI, 0.354–0.667) for CDAI tertiles 2 and 3, respectively (*p* for trend <0.001). Moreover, this correlation remained consistent in the fully modified model, and the trend was robust.

**Table 2:** Logistic regression analysis on the association between CDAI and SO.

	Model 1		Model 2^a^		Model 3^b^	
	OR (95% CI)	*P*	OR (95% CI)	*P*	OR (95% CI)	*P*
CDAI (continuous)	0.928 (0.891–0.965)	< 0.001^*^	0.923 (0.882–0.963)	< 0.001^*^	0.921 (0.883–0.956)	< 0.001^*^
CDAI (categories)						
Tertile 1	Reference		Reference		Reference	
Tertile 2	0.639 (0.476–0.855)	0.003^*^	0.594 (0.434–0.811)	0.001^*^	0.626 (0.463–0.842)	0.002^*^
Tertile 3	0.535 (0.392–0.723)	< 0.001^*^	0.498 (0.358–0.689)	< 0.001^*^	0.487 (0.354–0.667)	< 0.001^*^
*P* for trend	< 0.001^*^		< 0.001^*^		< 0.001^*^	

^*^Statistically significant (*P* < 0.05). CDAI, Composite Dietary Antioxidant Index; SO, sarcopenic obesity; OR, odds ratio; CI, confidence interval.

a Model 2: adjusted for age, sex, race, marital status, and education.

b Model 3: model 2 + smoking status, drinking status, hypertension, diabetes, and BMI.

**Figure 2:**
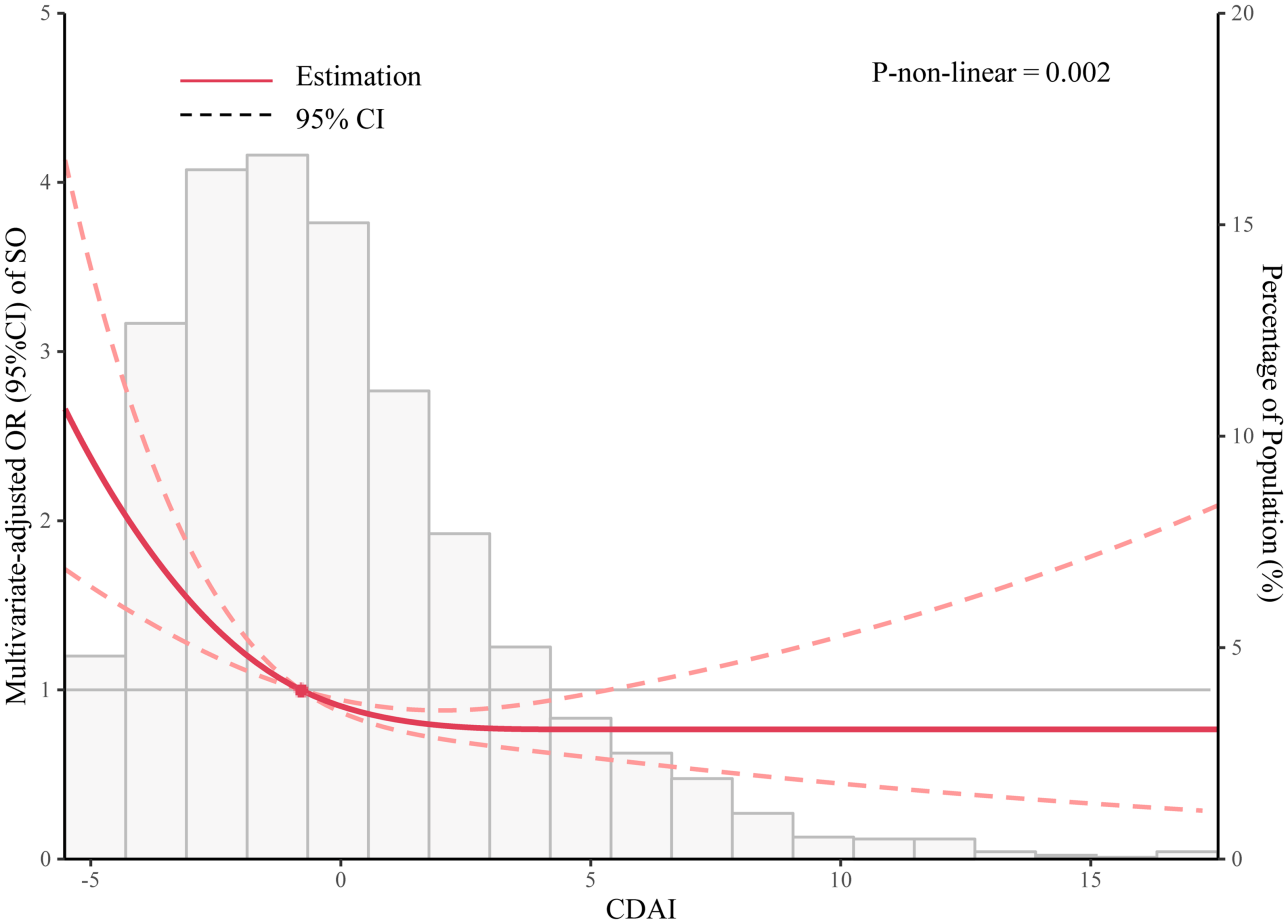
Restricted cubic spline of OR and 95% CI for the association between CDAI and SO.

Figure 2 shows the restricted cubic spline of OR and the 95% CI for the association between CDAI and SO. We discovered an L-shaped relationship between CDAI and SO (*P* for non-linearity = 0.002).

Table 3 shows the results of the subgroup analysis. The subgroup analysis findings demonstrated a reliable and enduring connection between CDAI and SO across various subgroups. Notably, no significant interactions were observed for sex, hypertension, or BMI, suggesting that the association was not dependent on these variables (all *p* for interaction >0.05). Nevertheless, the strength of the correlation between CDAI and SO was significantly affected by diabetes (*p* for interaction = 0.027). Individuals with diabetes faced an elevated risk in contrast to those without diabetes.

**Table 3:** Results of subgroup analyses were stratified by sex, hypertension, diabetes, and BMI.

Variables	OR	95% CI	*P*	*P* for interaction
Sex				0.163
Male	0.904	0.845–0.961	0.002^*^	
Female	0.933	0.878–0.987	0.019^*^	
Hypertension				0.176
No	0.957	0.887–1.028	0.246	
Yes	0.903	0.854–0.952	< 0.001^*^	
Diabetes				0.027^*^
No	0.943	0.899–0.986	0.012^*^	
Yes	0.809	0.706–0.911	0.001^*^	
BMI				0.062
< 25 kg/m^2^	0.938	0.882–0.994	0.034^*^	
25–30 kg/m^2^	0.880	0.821–0.939	< 0.001^*^	
≥ 30 kg/m^2^	1.102	0.873–1.259	0.280	

^*^Statistically significant (*P* < 0.05). Adjusted for age, sex, race, marital status, education, smoking status, drinking status, hypertension, diabetes, and BMI. Stratified variables themselves were not adjusted in the subgroup analysis.

Table 4 shows the logistic regression analysis of the association between CDAI and SO after removing outliers. The top 5% (117) and bottom 5% (117) of CDAI were removed. The relationship between CDAI and SO remained consistent across various models. In model 1, the OR of CDAI on SO was 0.914 [95% CI, 0.865–0.965]. Participants in the uppermost CDAI tertiles compared to those in the lowest CDAI tertiles had an increased susceptibility to SO [OR: 0.588 (95% CI, 0.423–0.811)]. In model 3, after adjusting for age, sex, race, marital status, education, smoking status, drinking status, hypertension, diabetes, and BMI, the OR was 0.905 (95% CI, 0.855–0.957). The ORs were 0.719 (95% CI, 0.525–0.980) and 0.552 (95% CI, 0.395–0.767) for CDAI tertiles 2 and 3, respectively (*p* for trend <0.001). Moreover, this correlation remained consistent in the fully modified model, and the trend was robust.

**Table 4:** Logistic regression analysis on the association between CDAI and SO after removing outliers.

	Model 1		Model 2^a^		Model 3^b^	
	OR (95% CI)	*P*	OR (95% CI)	*P*	OR (95% CI)	*P*
CDAI (continuous)	0.914 (0.865, 0.965)	0.001^*^	0.910 (0.856, 0.965)	0.002^*^	0.905 (0.855, 0.957)	< 0.001^*^
CDAI (categories)						
Tertile 1	Reference		Reference		Reference	
Tertile 2	0.725 (0.531, 0.987)	0.042^*^	0.690 (0.495, 0.957)	0.027^*^	0.719 (0.525, 0.980)	0.038^*^
Tertile 3	0.588 (0.423, 0.811)	0.001^*^	0.564 (0.397, 0.795)	0.001^*^	0.552 (0.395, 0.767)	< 0.001^*^
*P* for trend	0.001^*^		0.001^*^		< 0.001^*^	

^*^Statistically significant (*P* < 0.05). CDAI, Composite Dietary Antioxidant Index; SO, sarcopenic obesity; OR, odds ratio; CI, confidence interval.

a Model 2: adjusted for age, sex, race, marital status, and education.

b Model 3: model 2 + smoking status, drinking status, hypertension, diabetes, and BMI.

## 4 Discussion

To the best of our knowledge, this study represents the initial population-based retrospective cohort investigation into the correlation between CDAI and SO. Our findings indicate that CDAI exhibits a negative association with the likelihood of SO, even after accounting for potential confounding factors. Specifically, we observed an L-shaped relationship between CDAI and SO. Further subgroup analysis revealed that this phenomenon was particularly pronounced within the diabetic population. The sensitivity analyses verified the robustness of the results.

Various definitions of SO have been used in research studies, yet the establishment of diagnostic criteria and cutoff points remains elusive. Among these investigations, anthropometric indices, particularly BMI and waist circumference, have been predominantly utilized for evaluating obesity. Despite their practicality and lack of specialized or costly equipment, anthropometric measurements have demonstrated limited sensitivity in identifying obesity among older individuals. Originally, most articles defined and stratified obesity based on BMI values, most likely for its simple evaluation and wide utilization. Afterward, excess body fat was found, which may also notably lead *per se* to functional impairment and disability due to motor or cardiorespiratory complications. Therefore, fat mass was used in several studies implementing body composition analysis techniques. According to the European Society for Clinical Nutrition and Metabolism (ESPEN) and the European Association for the Study of Obesity (EASO) consensus statement, total fat mass was used to define obesity (2). To prevent the oversight of obesity in this population, the assessment of fat percentage appears more suitable, particularly for the evaluation of SO (17). The reported occurrence of SO varies from 2.75% to more than 20%, based on the utilized diagnostic standards and the techniques used for evaluating body composition (3, 18). In this study, SO was defined as the combination of excessive fat accumulation and low muscle mass, and the prevalence of SO was 20%.

Our findings indicated a negative correlation between CDAI and SO. The relationship might be related to the fact that SO is closely related to oxidative stress. Oxidative stress has been widely acknowledged as a significant contributor to the rate of aging and a crucial element in the aging process and pathological pathway. The imbalance between the production of antioxidants and pro-oxidants results in tissue and organ damage (19). The accumulation of reactive oxygen species (ROS) can induce the oxidation of DNA, proteins, carbohydrates, and lipids, ultimately leading to apoptosis and organ dysfunction. Diet, acting as an external influence, governed the redox status of plasma and provided defense against ROS and reactive nitrogen species. Antioxidants function by providing an electron to a free radical, which stabilizes it and reduces its reactivity, ultimately safeguarding the organism against the effects of oxidative stress (20). In order to preserve a stable biological redox equilibrium, antioxidants can eliminate oxidants, thereby averting oxidative stress (21). The consumption of external antioxidants halted inflammation, blocked atherosclerosis, countered insulin resistance, and alleviated oxidative stress.

The components of CDAI, including vitamin A, vitamin C, vitamin E, zinc, selenium, and carotenoids, played an important role in reducing stress-induced changes in oxidants. The evidence indicate that consuming higher levels of specific forms of vitamin A can enhance immune functioning and increase resistance to oxidative stress (22, 23). Possible outcomes of vitamin C encompassed boosting neutrophil levels through dietary consumption, reducing the generation of ROS while engulfing foreign particles, and impeding the oxidation of low-density lipoproteins (24). Different antioxidants and anti-inflammatory properties found in various members of the vitamin E family, including γ-tocopherol, δ-tocopherol, and γ-tocotrienol, play a significant role in the prevention and treatment of chronic diseases (25). Furthermore, zinc has been shown to function as an antioxidant in certain chemical systems. The elucidation of two mechanisms involved safeguarding sulfhydryl groups from oxidation and restraining the generation of reactive oxygen by transition metals. Studies conducted *in vitro* have shown that excessive levels of zinc display antioxidant properties in systems based on organelles and isolated cells (26). The preservation of the body’s optimal function of the intracellular enzyme glutathione peroxide and the extracellular protector selenoprotein P, which protects against oxidative stress, was aided by dietary antioxidants, such as selenium (27, 28). Furthermore, previous studies had suggested that carotenoids played a crucial role as essential precursors in the production of retinol, such as vitamin A. Carotenoids and their enzymatic derivatives served as antioxidants in lipid-rich surroundings (29).

To uncover the fundamental reality, we performed a subgroup analysis in this investigation to maximize the utilization of data. During the subgroup analysis, a noteworthy correlation was noticed between CDAI and the pre-established risk factors for SO. The findings suggested that individuals with diabetes faced an elevated risk in comparison to those without diabetes. Possibly, this could be because these people experience increased oxidative stress and consuming antioxidants from external sources seems to offer greater protection for those with elevated levels of ROS, whether it is innate or acquired (30). The findings of our study indicated that individuals with a high susceptibility to SO might experience greater advantages from consuming more antioxidants in their diet.

Nevertheless, it was important to recognize that there were specific constraints that needed to be acknowledged. Due to its cross-sectional design, the study was unable to establish a causal relationship between CDAI and SO. Additional studies of longer duration were required to investigate the time-dependent aspect of this correlation. Furthermore, even after accounting for various potential confounding factors, there was still a possibility that the outcomes were influenced by unmeasured variables. Moreover, it was important to exercise caution when generalizing the results to populations beyond the United States due to the study’s limitation to American participants.

## 5 Conclusion

The present study identified the L-shaped correlation between CDAI and SO in elderly participants’ demographics, following an adjustment for potential confounding factors. Our study indicated that individuals with a susceptibility to SO might experience advantages from consuming antioxidants in their diet.

## Data Availability

The original contributions presented in the study are included in the article/supplementary material, further inquiries can be directed to the corresponding authors.

## References

[1] Barazzoni R Bischoff S Boirie Y Busetto L Cederholm T Dicker D. Sarcopenic obesity: time to meet the challenge. Obes Facts. (2018) 11:294–305. doi: 10.1159/000490361, PMID: 30016792 PMC6189532

[2] Donini LM Busetto L Bischoff SC Cederholm T Ballesteros-Pomar MD Batsis JA. Definition and diagnostic criteria for sarcopenic obesity: ESPEN and EASO consensus statement. Clin Nutr. (2022) 41:990–1000. doi: 10.1016/j.clnu.2021.11.014 35227529

[3] Donini LM Busetto L Bauer JM Bischoff S Boirie Y Cederholm T. Critical appraisal of definitions and diagnostic criteria for sarcopenic obesity based on a systematic review. Clin Nutr. (2020) 39:2368–88. doi: 10.1016/j.clnu.2019.11.024, PMID: 31813698

[4] Atkins JL Wannamathee SG. Sarcopenic obesity in ageing: cardiovascular outcomes and mortality. Br J Nutr. (2020) 124:1102–13. doi: 10.1017/S0007114520002172, PMID: 32616084

[5] Van Aller C Lara J Stephan BCM Donini LM Heymsfield S Katzmarzyk PT. Sarcopenic obesity and overall mortality: results from the application of novel models of body composition phenotypes to the National Health and nutrition examination survey 1999-2004. Clin Nutr. (2019) 38:264–70. doi: 10.1016/j.clnu.2018.01.022, PMID: 29499977

[6] Wright ME Mayne ST Stolzenberg-Solomon RZ Li Z Pietinen P Taylor PR. Development of a comprehensive dietary antioxidant index and application to lung cancer risk in a cohort of male smokers. Am J Epidemiol. (2004) 160:68–76. doi: 10.1093/aje/kwh173, PMID: 15229119

[7] Luu HN Wen W Li H Dai Q Yang G Cai Q. Are dietary antioxidant intake indices correlated to oxidative stress and inflammatory marker levels? Antioxid Redox Signal. (2015) 22:951–9. doi: 10.1089/ars.2014.6212 25602689 PMC4376488

[8] Chen Y Tang W Li H Lv J Chang L Chen S. Composite dietary antioxidant index negatively correlates with osteoporosis among middle-aged and older US populations. Am J Transl Res. (2023) 15:1300–8. 36915799 PMC10006777

[9] Zhao L Sun Y Cao R Wu X Huang T Peng W. Non-linear association between composite dietary antioxidant index and depression. Front Public Health. (2022) 10:988727. doi: 10.3389/fpubh.2022.988727 36311643 PMC9609418

[10] Wu D Wang H Wang W Qing C Zhang W Gao X. Association between composite dietary antioxidant index and handgrip strength in American adults: Data from National Health and nutrition examination survey (NHANES, 2011-2014). Front Nutr. (2023) 10:1147869. doi: 10.3389/fnut.2023.1147869, PMID: 37063339 PMC10102380

[11] Wang M Huang ZH Zhu YH He P Fan QL. Association between the composite dietary antioxidant index and chronic kidney disease: evidence from NHANES 2011-2018. Food Funct. (2023) 14:9279–86. doi: 10.1039/D3FO01157G, PMID: 37772927

[12] Zheng H Yin Z Luo X Zhou Y Zhang F Guo Z. Associations between systemic immunity-inflammation index and heart failure: evidence from the NHANES 1999-2018. Int J Cardiol. (2023) 395:131400. doi: 10.1016/j.ijcard.2023.131400 37769969

[13] Wang L Yi Z. Association of the Composite dietary antioxidant index with all-cause and cardiovascular mortality: a prospective cohort study. Front Cardiov Med. (2022) 9:993930. doi: 10.3389/fcvm.2022.993930, PMID: 36267633 PMC9577254

[14] Maugeri A Hruskova J Jakubik J Kunzova S Sochor O Barchitta M. Dietary antioxidant intake decreases carotid intima media thickness in women but not in men: a cross-sectional assessment in the Kardiovize study. Free Radic Biol Med. (2019) 131:274–81. doi: 10.1016/j.freeradbiomed.2018.12.018, PMID: 30576781

[15] Baumgartner RN Koehler KM Gallagher D Romero L Heymsfield SB Ross RR. Epidemiology of sarcopenia among the elderly in New Mexico. Am J Epidemiol. (1998) 147:755–63. doi: 10.1093/oxfordjournals.aje.a009520, PMID: 9554417

[16] Baumgartner RN Wayne SJ Waters DL Janssen I Gallagher D Morley JE. Sarcopenic obesity predicts instrumental activities of daily living disability in the elderly. Obes Res. (2004) 12:1995–2004. doi: 10.1038/oby.2004.250, PMID: 15687401

[17] Ozkok S Aydin CO Sacar DE Catikkas NM Erdogan T Bozkurt ME. Sarcopenic obesity versus sarcopenia alone with the use of probable sarcopenia definition for sarcopenia: associations with frailty and physical performance. Clin Nutr. (2022) 41:2509–16. doi: 10.1016/j.clnu.2022.09.005, PMID: 36219979

[18] Prado CM Wells JC Smith SR Stephan BC Siervo M. Sarcopenic obesity: a critical appraisal of the current evidence. Clin Nutr. (2012) 31:583–601. doi: 10.1016/j.clnu.2012.06.010, PMID: 22809635

[19] van der Pol A van Gilst WH Voors AA van der Meer P. Treating oxidative stress in heart failure: past, present and future. Eur J Heart Fail. (2019) 21:425–35. doi: 10.1002/ejhf.1320, PMID: 30338885 PMC6607515

[20] Pizzino G Irrera N Cucinotta M Pallio G Mannino F Arcoraci V. Oxidative stress: harms and benefits for human health. Oxidative Med Cell Longev. (2017) 2017:8416763. doi: 10.1155/2017/8416763, PMID: 28819546 PMC5551541

[21] Demmig-Adams B Adams WW. Antioxidants in photosynthesis and human nutrition. Science. (2002) 298:2149–53. doi: 10.1126/science.1078002 12481128

[22] Meng XL Wang Y Wang HL Nie HH Cheng BJ Cao HJ. The association between essential trace element mixture and atherosclerotic cardiovascular disease risk among Chinese community-dwelling older adults. Environ Sci Pollut Res Int. (2022) 29:90351–63. doi: 10.1007/s11356-022-22066-0, PMID: 35869340

[23] Miller AP Coronel J Amengual J. The role of beta-carotene and vitamin a in atherogenesis: evidences from preclinical and clinical studies. BBA-Mol Cell Biol L. (2020) 1865:158635. doi: 10.1016/j.bbalip.2020.158635, PMID: 31978554 PMC7371525

[24] Moser MA Chun OK. Vitamin C and heart health: a review based on findings from epidemiologic studies. Int J Mol Sci. (2016) 17:17. doi: 10.3390/ijms17081328 PMC5000725 27529239

[25] Jiang Q. Natural forms of vitamin E: metabolism, antioxidant, and anti-inflammatory activities and their role in disease prevention and therapy. Free Radic Biol Med. (2014) 72:76–90. doi: 10.1016/j.freeradbiomed.2014.03.035, PMID: 24704972 PMC4120831

[26] Bray TM Bettger WJ. The physiological role of zinc as an antioxidant. Free Radic Biol Med. (1990) 8:281–91. doi: 10.1016/0891-5849(90)90076-U 2187766

[27] Xia Y Hill KE Li P Xu J Zhou D Motley AK. Optimization of selenoprotein P and other plasma selenium biomarkers for the assessment of the selenium nutritional requirement: a placebo-controlled, double-blind study of selenomethionine supplementation in selenium-deficient Chinese subjects. Am J Clin Nutr. (2010) 92:525–31. doi: 10.3945/ajcn.2010.29642, PMID: 20573787 PMC2921536

[28] de Lima-Reis SR Silva TA Costa LSA Volp ACP Rios-Santos F Reis EM. Serum levels of vitamin a, selenium, and better dietary total antioxidant capacity are related to lower oxidative DNA damage: a cross-sectional study of individuals at cardiovascular risk. J Nutr Biochem. (2022) 107:109070. doi: 10.1016/j.jnutbio.2022.109070, PMID: 35644409

[29] Yabuzaki J. Carotenoids database: structures, chemical fingerprints and distribution among organisms. Database (Oxford). (2017) 2017:bax004. doi: 10.1093/database/bax004, PMID: 28365725 PMC5574413

[30] Black HS. A synopsis of the associations of oxidative stress, ROS, and antioxidants with diabetes mellitus. Antioxidants. (2022) 11:11. doi: 10.3390/antiox11102003 PMC9598123 36290725

